# MTMol-GPT: De novo multi-target molecular generation with transformer-based generative adversarial imitation learning

**DOI:** 10.1371/journal.pcbi.1012229

**Published:** 2024-06-26

**Authors:** Chengwei Ai, Hongpeng Yang, Xiaoyi Liu, Ruihan Dong, Yijie Ding, Fei Guo

**Affiliations:** 1 School of computer science and engineering, Central South University, Changsha, China; 2 Department of computer science and engineering, University of South Carolina, Columbia, South Carolina, United States of America; 3 School of Chinese Materia Medica, Beijing University of Chinese Medicine, Beijing, China; 4 Ministry of Education, Engineering Research Center for Pharmaceutics of Chinese Materia Medica and New Drug Development, Beijing, China; 5 Academy for Advanced Interdisciplinary Studies, Peking University, Beijing, China; 6 Yangtze Delta Region Institute (Quzhou), University of Electronic Science and Technology of China, Quzhou, China; Hunter College, The City University of New York, UNITED STATES

## Abstract

De novo drug design is crucial in advancing drug discovery, which aims to generate new drugs with specific pharmacological properties. Recently, deep generative models have achieved inspiring progress in generating drug-like compounds. However, the models prioritize a single target drug generation for pharmacological intervention, neglecting the complicated inherent mechanisms of diseases, and influenced by multiple factors. Consequently, developing novel multi-target drugs that simultaneously target specific targets can enhance anti-tumor efficacy and address issues related to resistance mechanisms. To address this issue and inspired by Generative Pre-trained Transformers (GPT) models, we propose an upgraded GPT model with generative adversarial imitation learning for multi-target molecular generation called MTMol-GPT. The multi-target molecular generator employs a dual discriminator model using the Inverse Reinforcement Learning (IRL) method for a concurrently multi-target molecular generation. Extensive results show that MTMol-GPT generates various valid, novel, and effective multi-target molecules for various complex diseases, demonstrating robustness and generalization capability. In addition, molecular docking and pharmacophore mapping experiments demonstrate the drug-likeness properties and effectiveness of generated molecules potentially improve neuropsychiatric interventions. Furthermore, our model’s generalizability is exemplified by a case study focusing on the multi-targeted drug design for breast cancer. As a broadly applicable solution for multiple targets, MTMol-GPT provides new insight into future directions to enhance potential complex disease therapeutics by generating high-quality multi-target molecules in drug discovery.

## Author summary

Advancing drug discovery with the vast scale of possible structures of drug-like compounds (between 10^23^ and 10^60^) relies on Artificial Intelligence (AI). The process of generating SMILES has been accomplished by using language models like the Generative Pre-trained Transformer (GPT) model. In the meantime, regulating multiple targets to achieve the desired physiological responses is booming for treating complicated diseases. However, the recent deep generative models focus on single-target drug generation, and single-target drugs have limitations, such as requiring higher doses and causing mutations. For example, activation of Src kinase in certain breast cancer cells leads to EGFR phosphorylation and downstream effects. Consequently, designing multi-target molecules against multi-target simultaneously is highly required. Thus, we have proposed an upgraded GPT model MTMol-GPT with generative adversarial imitation learning for generating high-quality multiple targets drug-like compounds. For example, MTMol-GPT generates novel drugs targeting protein pairs such as DRD2 and HTR1A, EGFR and Src. As a broadly applicable tool, MTMol-GPT provides a rapid and accurate method for advancing drug discovery, ensuring the efficient generation of high-quality multi-target drugs.

## Introduction

De novo drug design generates novel compounds with specific pharmacologic and chemical properties, that can manifest a formidable binding affinity to a specific protein target. To date, the scale of possible structures of drug-like compounds is between 10^23^ and 10^60^ [1–3]. The discovery of optimal molecules can be framed as either a search for molecules from an enumerated library or a generation of novel molecules. Traditional drug design works utilize high-throughput technology [4] and conduct virtual screening [5] from existing molecules. Although traditional single-target drug design methods have led to promising results in filtering out the desired molecules. However, the models prioritize a single target drug generation for pharmacological intervention, neglecting the complicated inherent mechanisms of diseases, and influenced by multiple factors.

Indeed, given the wide array of disease targets, including targets such as EGFR and Src associated with breast cancer [6], it is evident that there is a compelling need to design multi-target molecules for treating complicated diseases. However, generating new molecules with biological activities toward dual-specific targets remains an extremely difficult challenge.

Recently, the development of deep generative methodologies has facilitated the generation of novel molecules within a vast chemical space [7–10], where molecules could be represented as Simplified Molecular-Input Line-Entry System (SMILES) [11] strings as input into sequential models such as Recurrent Neural Networks (RNN) [12–15], AutoEncoder (AE) [16, 17], and Generative Adversarial Networks (GAN) [18, 19]. Molecular generation with SMILES can parsimoniously describe the molecular structure in a sequence and is considered a text generation task [20–23]. Thus, a novel task that translates between SMILES and natural language has been proposed by using language models like the Generative Pre-Trained Transformer (GPT) model. Such models pre-trained to learn chemical structures from a large molecular dataset to generate new molecules of desirable properties and activity [20, 21, 24–27]. For example, the study proposed by Flam-Shepherdflam et al. [24] shows that language models efficiently learn complex molecular distributions. Concurrently, Olivecrona et al. [21] conducted experiments on designing active molecules against the dopamine type 2 (DRD2) receptor, and Popova et al. [20] designed molecules are active against the Janus protein kinase 2 (JAK2) receptor. Therefore, GPT variant techniques have been effectively employed to design de novo molecules specific to a single target by utilizing target-specific molecular substrates as input [28].

The potential of Reinforcement Learning (RL) uncovering new possibilities [29–31] has been introduced as an efficient solution to configure the chemical space for specific properties automatically [20, 21, 25–27]. These methods specially designed reward functions for a generative model and treated the score as a reward to optimize the model [25, 27]. Also, the RL policy is trained to add atoms and bonds to a molecular graph representation with Graph Neural Networks (GNNs) [32], which benefits from a straightforward mapping between molecules and their graph representations, simplifying the definition of state and action spaces with clear Markovian dynamics [33, 34]. However, the challenge of training GNNs and their limited use of large textual datasets about molecular structures and properties emphasizes the importance of developing advanced methods to address these issues [35, 36].

The Inverse Reinforcement Learning (IRL) [37–40] facilitates decision-making in complex problems by learning a reward function based on expert trajectories [41–43]. For example, Agyemang et al. [41] employed an RNN to learn the reward function directly and incorporated the guided reward learning method [42] during the RNN training process. Although IRL facilitates decision-making by learning a reward function, it can be computationally intensive and inefficient. In contrast, to bypass this problem, Ho et al. [43] introduce Generative Adversarial Imitation Learning (GAIL), which uses a discriminator to guide the actor in emulating expert behavior.

**Fig 1:**
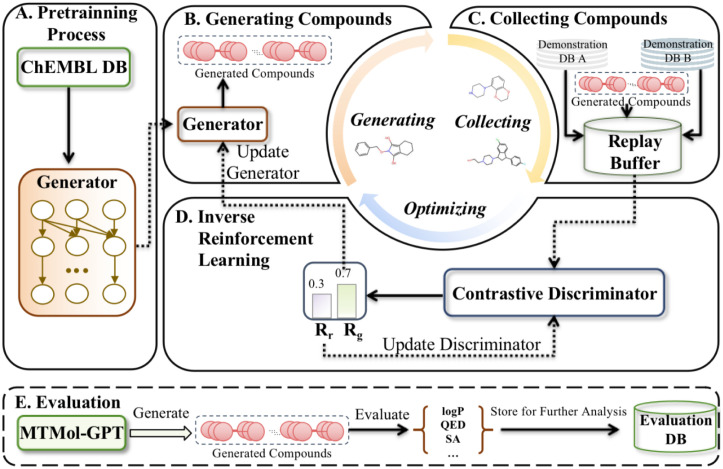
The overview workflow of MTMol-GPT. A) The transformer-based generator is pre-trained on ChEMBL database. B) The molecules are generated by the generator. C) The molecules toward different proteins as demonstrations and generated molecules are stored in a replay buffer. D) The output of the contrastive discriminator is utilized to update the generator and discriminator. E) The optimized generator is applied to generate molecules that will be used to evaluate the quality of the model.

Herein, we present a novel computational model named Generative Adversarial Imitation Learning integrated Generative Pre-trained Transformers for Multi-Target MOLecular generation (MTMol-GPT). As depicted in Fig 1, To take advantage of the richness of text-based representations for molecules and inspired by GPT, we first pre-train transformer neural networks on the ChEMBL database to guarantee that the generated sequence will be a valid drug-like structure compliant with the SMILES and Self-referencing embedded strings (SELFIES) [44]. We further develop a dual-target molecular sequence generation system through the GAIL algorithm. In addition, a replay buffer was utilized to store the molecules from different target types, and a dual contrastive discriminator was applied to estimate the relative realness between generated and real molecules, which was used as a reward to train the generator. Utilizing MOSES [45], MTMol-GPT shows state-of-the-art performance compared with other methods. The results for generating new molecules toward DRD2 and HTR1A, EGFR and Src showed that the proposed MTMol-GPT is capable of generating novel chemical matter specifically designed to hit a dual target of interest. Moreover, the molecular docking and pharmacophore mapping experiments enhanced and visualized the generated molecules’ excellent performance on both target proteins. In conclusion, our proposed MTMol-GPT model is a valuable tool for de novo drug design and has the potential to dramatically accelerate the drug optimization time.

## Results

### A workflow of MTMol-GPT

MTMol-GPT extends GAIL for multiple targets molecular generation tasks and is built on a GPT model. It involves five key steps: pre-training, generating compounds, collecting compounds in a replay buffer, updating the generator and discriminator, and evaluating the model (Fig 1). More details can be found in the Materials and Methods Section Materials and methods.

Unlike regular generative models, we utilize GAIL to optimize the model, introducing a contrastive discriminator to calculate rewards for generated sequences. The quality of our molecules was evaluated and outperformed by comparing results with SOTA models on molecular generation tasks. In addition, molecular docking and pharmacophore mapping experiments were conducted to assess binding capabilities, and we improved validity by introducing SELFIES representation. Overall, our model demonstrates high similarity to training data and shows potential for dual-targeting. MTMol-GPT offers superior performance, generating diverse multi-target molecules for drug discovery, and addressing diseases affected by multiple factors.

### Assessment of molecular generation

Progress in pharmacology emphasizes targeting the DRD2 and HTR1A receptors for improved neuropsychiatric interventions [46]. These crucial G protein-coupled receptors impact neurological and psychiatric disorders. Targeting DRD2 influences motor control, reward processing, and decision-making in conditions like Parkinson’s disease and schizophrenia [47]. HTR1A, a key player in serotonin regulation, is implicated in mood and anxiety disorders, preserving mood stability and cognitive functioning [48]. Focusing on these receptors in drug design shows promise for transformative advancements in mental health therapeutics, necessitating further research and development efforts [46]. Thus, in our study, extensive experiments were conducted on these receptors with MTMol-GPT.

#### Evaluating generation of molecules with a specific attribute

Drawing upon widely accepted paradigms in the realm of cheminformatics, it is frequently observed that molecules displaying analogous structural characteristics often exhibit comparable biological activities [23]. This assertion is substantiated via the rigorous scrutiny of molecular structures synthesized, utilizing pertinent reference datasets as comparators. Consequently, with the employment of DRD2 and HTR1A test sets serving as these reference compendiums, we utilized MOSES metrics (details refer to Section MOSES metrics) to systematically evaluate the bioactivity potential of the generated molecules towards both DRD2 and HTR1A.

With the booming of GPT, molecular string representations were originally developed for database storage and molecule identification and have found a renaissance. In the intricate field of de novo drug design, there exists a diversity of molecular string representations, including but not limited to SMILES and SELFIES [44]. While the former fails to consistently represent valid molecular structures, the latter inherently guarantees the portrayal of a legitimate chemical graph with each string. Thus, contemplating this inherent consistency, we put forward a modified version of our model that employs SELFIES as the primary input, a variant we have named SF-MTMol-GPT.

As summarized in Table 1, for targeting DRD2, the validity of MTMol-GPT and SF-MTMol-GPT achieved 0.87 and 1 respectively. In addition, MTMol-GPT reached the lowest FCD score of 4.82 and the highest value for Frag of 0.98. For targeting HTR1A, the MTMol-GPT achieved the best performance of Unique@100 of 0.99, Unique@1000 of 0.95, Novel of 0.99, Frag of 0.97, and lowest FCD score of 4.79. Therefore, MTMol-GPT and SF-MTMol-GPT are efficient in generating new molecules with specific pharmacological properties. For comparison with the current SOTA methods, the results are detailed in Tables A and B in S1 Text.

**Table 1:** Comparison of MTMol-GPT and SF-MTMol-GPT in single target task by using MOSES metrics.

Metrics	DRD2		HTR1A	
	MTMol-GPT	SF-MTMol-GPT	MTMol-GPT	SF-MTMol-GPT
Valid	0.8700	**1.0000**	0.8089	**1.0000**
Unique@1000	0.9890	0.9380	**0.9900**	0.9620
Unique@10000	0.9345	0.7970	**0.9462**	0.8328
Novel	0.9870	0.9668	**0.9887**	0.9683
IntDiv	0.8405	**0.8557**	0.8390	0.8550
*FCD(DRD2)	**4.8212**	5.5787	8.5087	10.3727
SNN(DRD2)	0.4346	**0.4385**	0.3574	0.3251
Frag(DRD2)	**0.9818**	0.9547	0.9443	0.9175
Scaff(DRD2)	0.6553	**0.6827**	0.1502	0.1782
*FCD(HTR1A)	8.5154	10.2562	**4.7907**	5.8430
SNN(HTR1A)	0.3711	0.3386	0.4192	**0.4259**
Frag(HTR1A)	0.8830	0.8139	**0.9654**	0.9061
Scaff(HTR1A)	0.2141	0.1345	0.4100	**0.5660**

* denotes that the lower score in this metric is better. Bolded values denote the best performance for each metric.

In addition, another noteworthy instance arises when exclusively DRD2 molecules are deployed as inputs for the single discriminator. In such a scenario, the molecules that are produced fare considerably better on metrics when DRD2 molecules are used as benchmark data. This pattern prevails regardless of whether the MTMol-GPT or SF-MTMol-GPT is employed. Analogous conclusions were reached when the HTR1A molecule was used as the input. These findings, thus, substantiate the ability of our solitary discriminator to direct the generator towards creating molecules with diverse targeting. Moreover, these findings also suggest that when molecules from both targets are utilized as inputs, our models equipped with dual discriminators (details can be found in Section Dual contrastive discriminator) yield balanced results on the metrics associated with both targets.

#### Evaluating generation of molecules for multiple targets

For the multi-target molecular generation task, we evaluated MTMol-GPT and SF-MTMol-GPT by comparing them to three state-of-the-art (SOTA) multi-target molecular generation models: DLGN [23], RationaleRL [49], and CMolRNN [50]. DLGN [23] utilized adversarial training and reinforcement learning for dual target molecule generation. RationaleRL [49] excelled in multi-property optimization, generating molecules based on combined rationales. CMolRNN [50] employed a generative graph model for various drug design problems. MTMol-GPT and SF-MTMol-GPT are applied to generate single-target molecules toward DRD2 or HTR1A, respectively. The MOSES metrics have been applied here.

For a fair comparison, each model (DLGN, RationaleRL, and CMolRNN) was limited to generate 30, 000 molecules targeting DRD2 and HTR1A, and the MOSES metrics are implemented for evaluation.

As summarized in Table 2, the validity value of MTMol-GPT is higher than the SOTA model DLGN, demonstrating the superiors of the transformer-based generator. One possible reason may be transformer-based generator can better learn semantic information about molecules than the RNN-based model. While comparing with CMolRNN and RationaleRL, our model MTMol-GPT and DLGN have lower validity values. This is expected due to CMolRNN, and RationaleRL are graph-based models, which can learn structural information of molecules to overcome the SMILES not always a valid issue to improve the validity.

**Table 2:** Comparison of MTMol-GPT and other methods by using MOSES metrics.

Metrics	CMolRNN	RationaleRL	DLGN	MTMol-GPT	SF-MTMol-GPT
Valid	0.9796	0.998	0.7801	0.8453	**1.0000**
Unique@1000	0.4990	**0.999**	0.991	0.993	0.9550
Unique@10000	0.2628	**0.9998**	0.9718	0.9545	0.8140
Novel	0.6388	**1.000**	0.9982	0.9836	0.9322
IntDiv	0.8248	0.8275	0.8352	0.8349	**0.8500**
*FCD(DRD2)	7.2599	15.802	6.5719	**5.6181**	6.0967
SNN(DRD2)	**0.4699**	0.2869	0.3791	0.4051	0.4132
Frag(DRD2)	0.8219	0.7311	0.9528	**0.9731**	0.9617
Scaff(DRD2)	0.2207	0.0039	0.2498	0.4607	**0.6361**
*FCD(HTR1A)	6.7187	14.9645	6.5198	**5.3975**	6.5328
SNN(HTR1A)	**0.4741**	0.2812	0.3911	0.4140	0.4039
Frag(HTR1A)	0.8777	0.7338	0.9448	**0.9545**	0.9009
Scaff(HTR1A)	0.2591	0.0053	0.2678	**0.3915**	0.3582

* denotes that the lower score in this metric is better. Bolded values denote the best performance for each metric.

Although the graph-based models have better results, as seen in Table 2, the validity value of SF-MTMol-GPT is 1. SF-MTMol-GPT is our variant model, which achieved the best results in validity due to the robust molecular string representation with SELFIES. On the other aspect, the application of SELFIES can also cause some adverse effects. Comparing the results in uniqueness and novelty, MTMol-GPT has a higher scorer than SF-MTMol-GPT, which means that the SELFIES grammar leads to more memorization of the training dataset in language models.

Moreover, the similarity metrics were also applied to evaluating the generated dual-targets molecules with DRD2 and HTR1A test sets, such as FCD, Frag, Scaff, and SNN (More details in Section MOSES metrics). Our model MTMol-GPT achieved the best performance on FCD, Frag, and Scaff. Although CMolRNN showed the best SNN values, notably, our models (MTMol-GPT and SF-MTMol-GPT) consistently outperformed graph-based models such as RationaleRL, CMolRNN, and language model DLGN.

Specifically, MTMol-GPT achieved the lowest FCD values of 5.40. Compared with RationaleRL and CMolRNN, MTMol-GPT decreased the FCD value by 9.57 and 1.32, respectively. Additionally, MTMol-GPT and SF-MTMol-GPT exhibited superior performance, achieving the highest values over 0.95 on Fragment similarity and Scaffold similarity. The Frag values of MTMol-GPT and SF-MTMol-GPT are close in both DRD2 and HTR1A test sets, indicating molecules generated by our models and reference sets have similar fragments and can generate multiple targets molecules. In detail, with the DRD2 test sets, MTMol-GPT and SF-MTMol-GPT have the Frag values of 0.97, and 0.96, respectively. Also, for HTR1A test sets, our models outperformed compared three models with an average increase up to 0.20% (as shown in Table 2).

Furthermore, comparing Scaff similarity metric, SF-MTMol-GPT outperforms DLGN by 0.40 for DRD2, and 0.36 for HTR1A, which demonstrates the generated molecules are highly similar to known molecules of DRD2 and HTR1A on the scaffolds.

The results evaluated by MOSES and various similarity metrics demonstrated that our model signifies its reliability and accuracy in generating multiple targets molecules.

#### Validating physicochemical properties of generated molecules

Properties of molecules such as drug-likeness (QED), the octanol-water partition coefficient (LogP), and synthetic accessibility (SA) are important indicators to evaluate the quality of generated molecules [23]. Specifically, QED varies from 0 to 1 and estimates how likely a molecule is a viable candidate for a drug. The measurement of LogP shows the hydrophilic or hydrophobic of a molecule. SA is a score ranging from 1 to 10, and the higher score refers to the harder to synthesize a given molecule.

**Fig 2:**
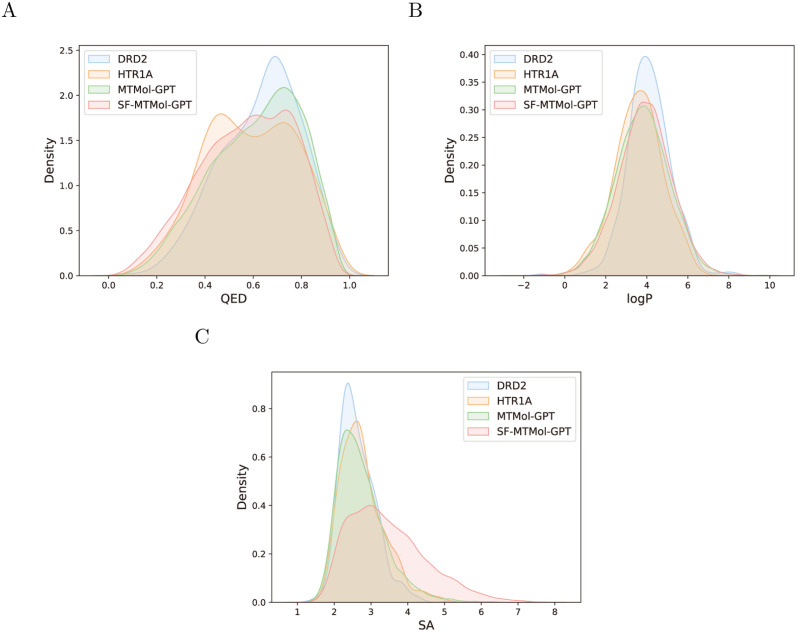
Distributions of molecular properties with optimized model. A) QED, B) LogP, and C) SA score distributions for DRD2, HTR1A, and molecules generated by the optimized MTMol-GPT and SF-MTMol-GPT.

In this section, experiments have been conducted to evaluate the MTMol-GPT/SF-MTMol-GPT can well learn the distribution of molecular properties. We calculate the above-mentioned properties of the ChEMBL dataset, DRD2 test dataset, HTR1A test dataset and 10, 000 valid and unique molecules respectively sampled by our model. We compared the distributions of molecules generated by the optimal model after fine-tuning with those of DRD2 and HTR1A test sets (as shown in Fig 2). In addition, we compared the property distributions of molecules generated by the pre-trained model with the ChEMBL dataset (Fig A in S1 Text). For both experiments, MTMol-GPT and SF-MTMol-GPT were set to generate 10, 000 valid molecules, respectively.

As shown in Fig 2, the property distributions of molecules generated by the optimized MTMol-GPT and SF-MTMol-GPT and molecules from DRD2 and HTR1A datasets are compared. As we can see, the property distributions of the generated molecules are always at the intersection region of the different target molecules’ property distributions. It is worth noting that the model has no conditions on properties. Nevertheless, from the visualization of the generated molecule structure (detailed in Figs B-E in S1 Text), the generator can still learn the property distributions of the molecules very well. In addition, the result showed applying GAIL can optimize the generator to generate molecules consistent with the multi-target molecule distributions by comparing the distribution before and after optimization.

### Assessment of targeting ability

To authenticate the potential drug-likeness of the synthesized compounds, we undertook two rigorous examinations. Initially, we employed molecular docking to explore the affinity of our constructed drug-like compounds with DRD2 and HTR1A targets. Subsequently, through the utilization of pharmacophore mapping, we initiated an exhaustive evaluation to determine if the molecular architectures conceived by MTMol-GPT could harmonize with the essential pharmacophores of DRD2 and HTR1A targets, thereby affirming their potential therapeutic effectiveness.

#### Molecular docking

We performed molecular docking to calculate the binding affinity of our generated molecules to validate whether they can target multiple proteins of interest as potential ligands. The structures of our target proteins DRD2 (PDB ID: 6LUQ) and HTR1A (PDB ID: 7E2Y) were obtained from the Protein Data Bank (PDB) [51]. We generated 1, 000 molecules with MTMol-GPT, and extracted 1000 inactive molecules for DRD2 and HTR1A from ExCAPE-DB, respectively. Next, we randomly selected the same amount of active molecules from datasets for DRD2 and HTR1A.

**Fig 3:**
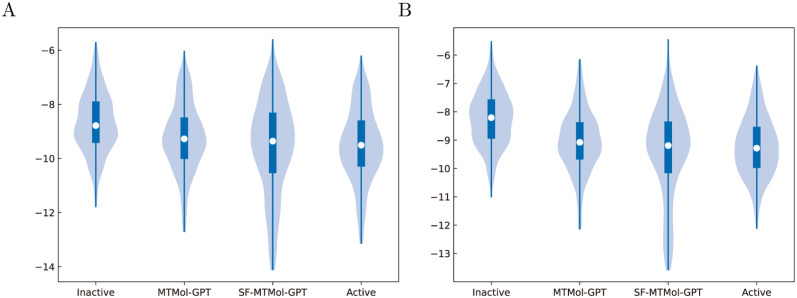
Distributions of docking score for DRD2 and HTR1A. A) DRD2. B) HTR1A.

After collecting the data, AutoDock Vina was used to perform molecular docking (details refer to Section AutoDock Vina), and the results of Docking scores (Ds) were shown in Fig 3. As for DRD2, the root mean square deviation (RMSD) value of ligand haloperidol between the experimental conformation in complex and our redocked pose is 0.38Å. And the RMSD for serotonin in HTR1A is 0.68Å. Both RMSD values are smaller than 2Å, which means our docking settings are reliable for these two receptors (see Fig F in S1 Text). The results indicate that molecules generated by both MTMol-GPT and SF-MTMol-GPT have outstanding performance on both DRD2 and HTR1A targets. Comparing the results for the two targets, the median docking score of generated molecules is smaller than that of inactive molecules. And the distribution of scores is also similar to that of active molecules. This indicates that the docking performance of the generated molecules on the two targets respectively is consistent with that of the existing active molecules.

**Fig 4:**
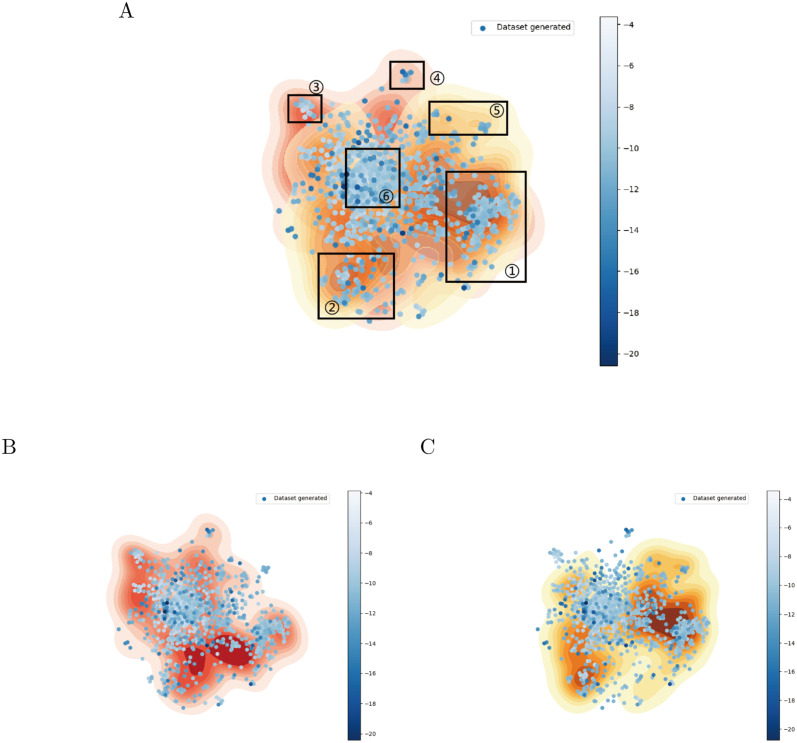
Visualization of molecule distributions from generated, DRD2, and HTR1A datasets using t-SNE. A) Molecules from generated, DRD2 and HTR1A testing datasets. B) Molecules from generated and DRD2 testing datasets. C) Molecules from generated and HTR1A testing datasets. The density plot shows DRD2 and HTR1A target distributions: red for DRD2, yellow for HTR1A. Generated molecules are colored by their docking values to both targets, with darker blue indicating stronger binding (lower values).

In addition, molecules generated by MTMol-GPT are capable of dual-targeting DRD2 and HTR1A, which was validated through the comparison with dense regions (Fig 4A). Therefore, we used the generated 1, 000 molecules with MTMol-GPT and compared them with the molecules from DRD2 (Fig 4B) and HTR1A (Fig 4C) testing datasets. To enhance the visualization of molecular distribution, we generated density maps with t-SNE and dimensionality-reduced coordinates for both DRD2 and HTR1A target molecule datasets.

In Fig 4A, while most generated molecules reside in the shared area of the two targets, a few also gather in distinct regions with higher color intensity, and lower docking values. Specifically, Fig 4B and 4C illustrates that the generated molecules primarily cluster within the regions of known DRD2- and HTR1A-targeting molecules. By overlapping these two targets’ separate distributions, we found that our generated molecules lay in the overlap regions, indicating the similarity between the generated molecules and known ligands.

To gain an insight into diverse molecules, we defined six clustering regions in total and examined molecules from these specific areas, including the overlapping zones (regions 1 and 2) of DRD2 and HTR1A, areas unique to DRD2 (regions 3 and 4), areas unique to HTR1A (region 5), and zones outside the central distribution of both targets (region 6). Molecules in undefined regions were excluded due to indistinct clustering. After a pre-selection process that involved filtering out molecules beyond the QED, logP, and SA value ranges defined by the DRD2 and HTR1A training sets, we selected one molecule with the lowest average docking values of two targets from each region (details in Table C in S1 Text).

**Fig 5:**
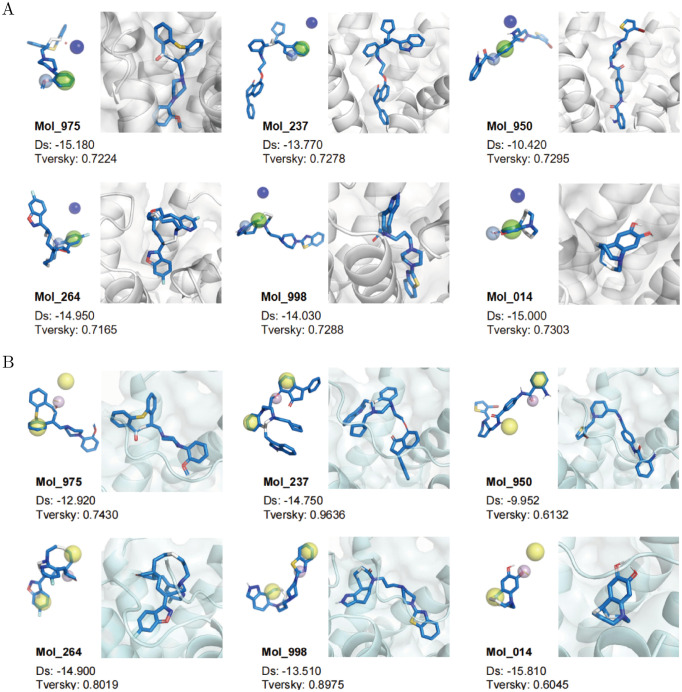
Molecular docking and pharmacophore mapping results of representative molecules. A) The docking results of DRD2 target. B) The docking results of HTR1A target. Mol_975, Mol_237, Mol_950, Mol_264, Mol_998, and Mol_014 are the representative molecules of 6 regions, respectively. The Docking score (Ds) lower is better, and Tversky is the overlapping ratio with target pharmacophore models (higher is better). As for pharmacophores, the deep blue ball is a cation center, the light blue ball is a hydrogen bond donor, the green ball is a merged aromatic ring, purple ball is a hydrogen bond acceptor, and yellow balls are hydrophobic centers.

Then we displayed the best docking conformation of these six representative molecules towards both the DRD2 and HTR1A targets in Fig 5A and 5B. Compared with their original ligands haloperidol and serotonin −9.68 and −6.06, respectively, our generated molecules can reach lower docking scores and better binding potential. Moreover, the generated molecules displayed various 3D conformations, further indicating that our model is capable of generating diverse and novel molecules that target two proteins simultaneously. Meanwhile, we analyzed the non-covalent interactions between protein receptors and docked molecules by PLIP [52]. DRD2 crystal structures with different ligands own the hydrophobic interaction with Asp114 and pi-stacking with Trp386 (see 6LUQ and 6CM4 Fig Ga in S1 Text). As for our molecules generated by MTMol-GPT, we can also find some of these key interactions from the best docking poses(see Fig Gb in S1 Text). For example, Mol_975 also forms the hydrophobic interactions with Leu94, Phe110, and Asp114, while Mol_237 contributes to Leu94, Phe110, and Val115. Other representative molecules rely on similar interactions to bind with DRD2. Likewise, similar interactive residues in HTR1A are captured to form hydrophobic interactions when binding with distinct molecules, including Tyr96, Lys119, and Asn386 (see Fig H in S1 Text).

#### Pharmacophore mapping

Pharmacophores are spatial chemical features to describe the interaction patterns of a protein-ligand pair. These feature types involve the hydrophobic centers, hydrogen bond donors, hydrogen bond acceptors, cationic centers, and so on. Furthermore, to validate the efficacy of the molecules generated by MTMol-GPT, we analyzed whether their structures can match the pharmacophore models of DRD2 and HTR1A targets by Align-it [53].

We first extracted the DRD2 pharmacophore model from reference [47], which was initiated on ligand R-NPA and adjusted with a set of active compounds. This pharmacophore model contains a merged aromatic ring, a hydrogen bond donor, and a positive charge center, which are represented in green, light blue, and deep blue in Fig 5A, respectively. Most of our generated molecules can match two out of three pharmacophores, as shown in Fig 5A. Similarly, the HTR1A pharmacophore model involves two aromatic and lipophilic groups (shown in yellow) and a hydrogen bond acceptor (in purple) [48]. In Fig 5B, all six representative molecules can match the pharmacophores of HTR1A generally. For instance, the Mol_237 generated by MTMol-GPT exhibited a high-level match to HTR1A pharmacophores with a Tversky score of 0.9636.

To some extent, these dual-targeting molecules display substructures that match the pharmacophores of two targets simultaneously and exhibit some individual features of each target alone. This indicates that MTMol-GPT can generate molecules targeting both DRD2 and HTR1A aware of their specific features, rather than just imitating the similar blocks between DRD2 and HTR1A ligands.

### Generalized analysis on dual-target drug design

MTMol-GPT has the generalization capability of de novo multi-target molecular generation for various target pairs. As reported, activation of Src kinase in some breast cancer cells leads to the phosphorylation of EGFR and causes the downstream effect [54]. Thus, we perform an experiment of de novo molecular design toward EGFR and Src targets to demonstrate that our model can be applied to generate various multi-target molecules. Firstly, the molecules toward EGFR and Src are collected from ExCAPE-DB. The invalid and repetitive molecules are removed, splitting into a training and test set with a ratio of 8 : 2, respectively. The training set is used to optimize the model, and the test set is utilized to verify the model’s performance. 1000 valid molecules are generated with trained MTMol-GPT.

**Fig 6:**
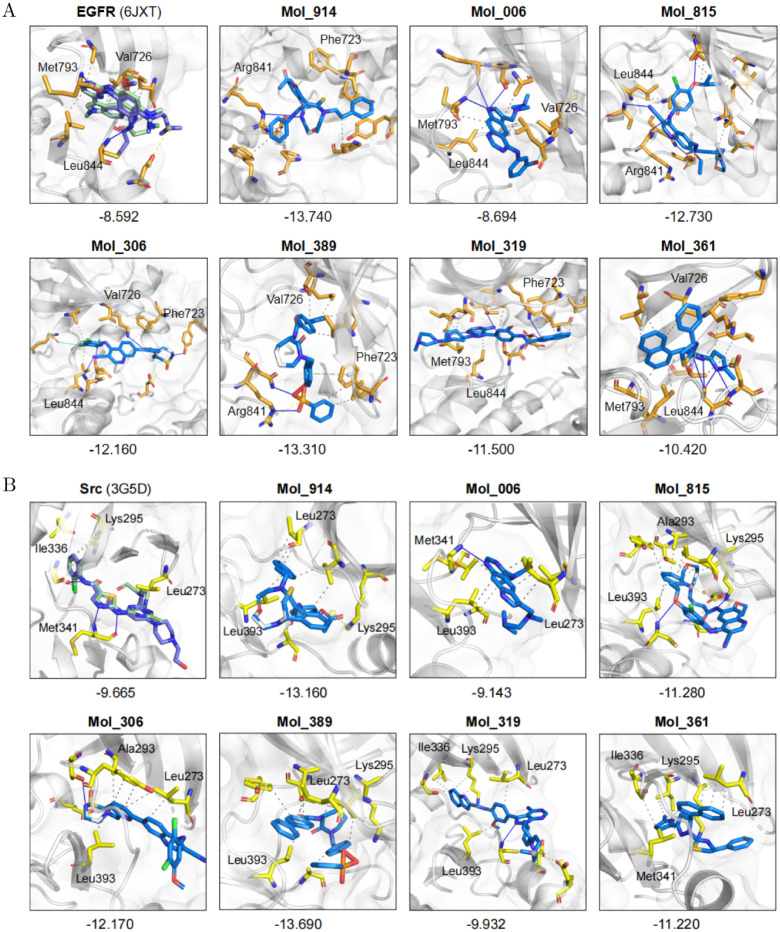
Protein-ligand binding interactions of representative generated molecules towards EGFR and Src. A) Protein-ligand binding interactions of representative generated molecules towards EGFR. B) Protein-ligand binding interactions of representative generated molecules towards Src. Mol_914, Mol_006, Mol_815, Mol_306, Mol_389, and Mol_361 are the representative molecules of 6 regions, respectively. Interactions are extracted from the best docking conformations and docking scores are shown. The first one shows original ligand in complex structure (in purple) and redocked pose (in palegreen).

We also conduct molecular docking and interaction analysis on the molecules generated by the EGFR and SRC targets. The method of selecting molecules is the same as in the above experiment. The t-SNE of generated molecules about EGFR and SRC dual targets, as well as the selected molecules (detailed in Figs A-C, and Table D in S1 Text). The reference complex structures are 6JXT (EGFR with ligand AZD9291) and 3G5D (Src with inhibitor dasatinib). We utilized the PLIP server to identify the non-covalent interactions between the protein and its ligands as well and marked several residues in Fig 6. It is observed that some residues are likely to form the same interactions with our generated molecules in their docking poses, compared with the complex. For instance, Leu844 and Val726 have hydrophobic interactions with the ligand in EGFR complex structure, while the Mol_006, Mol_306, and Mol_361 perform the same. Though Mol_914, Mol_815, and Mol_389 vary in molecular structures, they can form hydrogen bonds with Arg841 of EGFR target see Fig 6A). As for Src in Fig 6B, the ligand in a real complex can form hydrophobic interactions with Ile336, while Mol_319 and Mol_361 play the same. We can also find similar conditions on Leu273, Lys295, and some other key residues with different representative molecules.

In conclusion, extensive results demonstrate the generalization ability of MTMol-GPT to design multi-target ligands for different target proteins, with potential applications in drug discovery for complex diseases.

## Discussion

We present a novel model MTMol-GPT, which extends GAIL for molecular generation tasks and is built on a GPT model. Different from the regular generative models, we utilize GAIL to optimize the model. In the GAIL framework, a contrastive discriminator is introduced to calculate the reward of the generated sequence. The reward will guide the generator to generate multi-target molecules. To evaluate the quality of generated molecules, we compared the results of MOSES with SOTA models on the DRD2 and HTR1A molecular generation tasks. The results have shown that generated molecules by our model have lower FCD values and higher structural similarity-based metrics. Additionally, we conducted a docking experiment, using the docking scores of the molecule with the two target proteins to evaluate whether the molecule could bind to two targets of interest. Furthermore, due to the defects of SMILES, we also introduced the SELFIES representation of the molecule to improve the validity of generated molecules.

To validate that our model has the generation capability, we collected another different set of molecules for EGFR and Src to train our model. The generated molecules have high similarity with the molecules toward EGFR and Src in training data, indicating their possibility of dual-targeting. Moreover, there is still room for improvement in our proposed model. Although the SMILES-based representation can simplify the input and output of the model, lacking topological information leads to the emergence of low-quality and invalid molecules. Therefore, using molecular graphs to represent molecules should be used as prior knowledge to optimize language models. Furthermore, biochemical reactions are likely to be essential in multi-target molecule generation tasks, and introducing reactions in generative tasks is also a problem that can be further studied. In general, the proposed model can generate corresponding molecules for different combinations of protein targets, which helps develop multi-target drugs to a certain extent.

## Materials and methods

### Data collection

ChEMBL and ExCAPE-DB [55] datasets are used for pre-training and fine-tuning our model, respectively [23]. The ChEMBL dataset contains bioactive drug-like small molecules with recorded bioactivity information. Molecules extracted from this dataset serve as a pre-train dataset for the generator, and the model trained on this dataset can generate molecules satisfying general chemical properties.

To enhance the proficiency of MTMol-GPT in generating molecules targeting DRD2 and HTR1A, we utilized molecules targeting these receptors from ExCAPE-DB for model fine-tuning. Similarly, for improving molecule generation targeting EGFR and SRC in our generalized analysis, bioactive datasets from ExCAPE-DB targeting these receptors were also employed. This approach aims to boost the model’s capability to produce molecules with improved affinity and selectivity for these specific targets.

There are 344, 184 molecules from the ChEMBL as a pre-training dataset, 2, 156 molecules for the DRD2, 2, 787 molecules for the HTR1A, 3, 305 molecules for the EGFR, and 2, 036 molecules for the SRC. All datasets are summarized in Table 3.

**Table 3:** Details of ChEMBL and target molecule datasets.

Datasets	Train	Valid	Test
ChEMBL	344,184	-	-
DRD2 target	1,293	431	432
HTR1A target	1,672	557	558
EGFR target	2,645	-	660
SRC target	1,629	-	407

### Evaluation metrics

#### MOSES metrics

MOSES proposed by Polykovskiy et al. [45], containing a series of molecular evaluation metrics, such as Validity, Unique@*K*, Novel, and Internal diversity (IntDiv). Validity is the proportion of valid molecules in the generated molecules, which can be determined by the RDkit toolkit [56]. Unique@*K* refers to the proportion of unique molecules on all valid molecules. MOSES computes Unique@*K* for the first *K* = 1, 000 and *K* = 10, 000 valid molecules in the generated molecules. Novel denotes the proportion of generated molecules that do not appear in the training data set. IntDiv represents the chemical diversity within the generated valid molecules set.

In addition, similarity metrics are applied between the generated molecular set and the reference molecular sets. FréChet ChemNet distance (FCD) [57] indicates the similarity of chemical structures and bioactivities; Similarity to the nearest neighbor (SNN) refers to the average Tanimoto Similarity (Ts); Fragment similarity (Frag) is the cosine similarity in the BRICS segments [58]; Scaffold similarity (Scaff) is the cosine similarity in the Bemis-Murcko [59] scaffold of molecules.

#### AutoDock Vina

AutoDock Vina is the most widely used tool for docking and virtual screening [60] and is used for molecular docking in our study. Therefore, the physics-based docking score of molecules binding to targets was another evaluation metric. Regarding the docking score of known active molecules in the training set as a basis, if our generated molecules could obtain close or better docking affinity to the target proteins, generated molecules will likely interact with target proteins.

### MTMol-GPT

**Fig 7:**
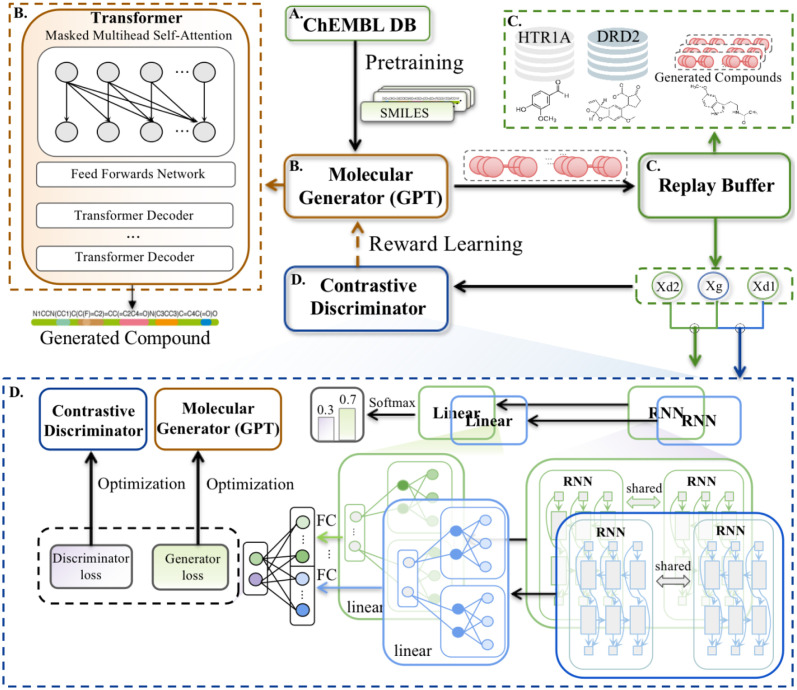
An overview of MTMol-GPT structure. A) ChEMBL database. B) The transformer-based generator is pre-trained on ChEMBL database. C) A replay buffer stores the molecules which toward different proteins as demonstrations and generated molecules. D) A dual contrastive discriminator updates the generator and discriminator.

The generation of multiple targets of new drugs with specific proteins is accomplished through the use of a machine learning algorithm named MTMol-GPT, which consists of four key modules in its learning architecture: pre-trained generator, replay buffer, dual contrastive discriminator, and GAIL. The overview of MTMol-GPT is illustrated in Fig 7.

#### Generator

The transformer-based generative model has shown state-of-the-art performance in language translation tasks, enabling better language embedding to simulate connections over longer distances [61]. This method has been used in various language processing-related tasks, which include commonsense reasoning, question answering, and textual entailment [62]. Meanwhile, transformers have two modules: encoder and decoder. The decoder contains the masked self-attention mechanism which is also beneficial for generating tasks and is known as GPT [61].

Therefore, we utilize a transformer-decoder with the masked self-attention mechanism as the generator in MTMol-GPT. As shown in Fig 7A and 7B, the generator is constructed by a 12-layer decoder and 8 attention heads. Each decoder block consists of a masked self-attention layer and a fully connected neural network.

For the generator pre-training process (details in Section Training process), SMILES was applied to represent a molecule as a sequence of tokens with the ChEMBL database (as shown in Fig 7A). We used ‘<’ character as the beginning token and ‘>’ as the end token. The beginning token and SMILES sequence of one molecule are taken as input. Moreover, SMILES with end tokens are the target for training the generator. Given a SMILES string *x* = {*x*_1_, *x*_2_, …, *x*_*T*_}, the model is trained following the loss function:

$$\begin{array}{c} L=-\sum_{t=1}^{T}\;logp_{\xi }\left(x_{t}|x_{<t}\right) \end{array}$$ (1)

where the parameters *ξ* are learned from the training set. In detail, to generate a sequence of SMILES tokens for a molecular generation, given the beginning character ‘<’, the GPT can generate a SMILES sequence according to a learned probability distribution. Then, the next character will be sampled from the probability distribution up to the end token or the sequence length to reach the maximum length.

#### Dual contrastive discriminator

According to previous research [23, 63], the contrastive discriminator estimates the relative realness between generated sequences and real sequences, which can significantly help conditional generation tasks. However, unlike the general discrimination task, the discriminator in our model is applied to determine that the generated molecules satisfy the properties of multiple targets simultaneously. The structure of the contrastive discriminator is shown in Fig 7D, which is mainly composed of two RNN networks.

Several targeted molecules are used as demonstration data for guiding molecule generation [23]. For each type of demonstration data, we use a module to output the feature to represent the generated and demonstration sequences. The module includes the RNN network and two connected layers. Firstly, applying the demonstration and generated sequences as the input to the RNN. Then, the fully connected layer was applied to the output of RNN. Next, the representations of demonstration and generated sequences are concatenated as the input to another fully connected layer. Finally, for multiple targets, we concatenate the output of each module, which uses a different type of demonstration sequence as input. The concatenated features are used for the multi-layer perception layers. The formula is as follows:

$$\begin{array}{cc} & h_{1}=Linear\left(RNN_{1}\left(x_{d_{1}}\right),RNN_{1}\left(x_{g}\right)\right), \end{array}$$ (2a)

$$\begin{array}{cc} & h_{2}=Linear\left(RNN_{2}\left(x_{d_{2}}\right),RNN_{2}\left(x_{g}\right)\right), \end{array}$$ (2b)

$$\begin{array}{cc} & p_{d},p_{g}=softmax\left(Linear\left(h_{1},h_{2}\right)\right) \end{array}$$ (2c)

where $x_{d_{1}}$ and $x_{d_{2}}$ represent the sequences of two types of demonstration data targeting different proteins, *x*_*g*_ denotes the generated sequence, and *p*_*d*_ and *p*_*g*_ are the prediction probabilities of the two target sequence and the generated sequence, respectively. Finally, the cross-entropy loss is utilized to optimize the contrastive discriminator. The loss would maximize the probability *p*_*d*_ for the real sequence. The generated sequence’s probability prediction *p*_*g*_ will be used as the reward signal to train the generator. More importantly, to fully use the sampled data and improve the stability of the model, the sequences collected are first sent to the replay buffer during the training process.

#### Generative adversarial imitation learning

GAIL was used for molecular optimization to generate molecules with desired properties. Consisting of the generator *G*_*ξ*_ with parameter *ξ* and the discriminator *D*_*ϕ*_ with parameter *ϕ*, the GAIL framework is applied to generate molecules toward multi-target. *G*_*ξ*_ is used to generate trajectories similar to experts, while *D*_*ϕ*_ regards the expert trajectories as positive samples and the trajectories generated by the generator as negative samples. Suppose expert trajectory *τ*_*E*_ ∼ *p*_*E*_, using the generator sample trajectory *τ*_*i*_ ∼ *G*_*ξ*_, the objective function of GAIL is as follows:

$$\begin{array}{c} {\text{min}}_{G_{\xi }}{\text{max}}_{D_{\phi }}E_{\tau_{E}∼p_{E}}\left[D_{\phi }\left(\tau_{E}\right)\right]+E_{\tau_{i}∼G_{\xi }}\left[1-D_{\phi }\left(\tau_{i}\right)\right] \end{array}$$ (3)

The Eq 3 is optimized to find the best generator and the best discriminator parameters. Usually, the parameters of the discriminator can be optimized by the derivative of the Eq 3, and the proximal policy optimization (PPO) [39, 64] algorithm can optimize the generator’s parameters.

To combine GAIL with the molecular SMILES sequence generation task, we define **S** as the space formed by different combinations of molecule SMILES characters and **A** as the space formed by the next character in the process of generating molecule sequence. Therefore, the trajectory *τ* of the SMILES sequence of length *T* is defined as follows:

$$\begin{array}{c} \tau =\{s_{1},a_{1},r_{1},\cdots ,s_{t},a_{t},r_{t},\cdots ,s_{T},a_{T},r_{T}\} \end{array}$$ (4)

where the *s*_*t*_ = *x*_1_*x*_2_ ⋯ *x*_*t*_ ∈ **S** and *a*_*t*_ ∈ **A** denote the state and action in *t*th step, and *r*_*t*_ is the reward obtained after performing action *a*_*t*_ in state *s*_*t*_. i.e. *s*_*t*_ is the SMILES sequence in the previous time *t*, *a*_*t*_ represents the behavior of adding SMILES characters to the model at time *t*, and *r*_*t*_ represents the reward obtained after the adding action at time *t*. Besides, we define the probability of an entire sequence *x* as:

$$\begin{array}{c} G_{\xi }\left(x\right)=\prod_{t=1}^{T}G_{\xi }\left(x_{t}|s_{t-1}\right) \end{array}$$ (5)

where *x* is the SMILES sequence, *T* is the SMILES length and *x*_*t*_ is the SMILES character at step *t*. Given a set of sequence trajectories **T** = {*τ*_1_, ⋯, *τ*_*i*_, ⋯, *τ*_*N*_} ∼ *G*_*ξ*_, the goal is to maximize the cumulative reward, as shown below:

$$\begin{array}{c} \bar{R}=E_{\tau ∼G_{\xi }}\left[R\left(\tau \right)\right]=\sum_{\tau }G_{\xi }\left(\tau \right)R(\tau ) \end{array}$$ (6)

Generally, the gradient ascending method can be used to optimize the Eq 6, which gradient is as follows:

$$\begin{array}{cc} \nabla \bar{R} & =\sum_{\tau }\nabla G_{\xi }\left(\tau \right)R(\tau )\\ & =\sum_{\tau }G_{\xi }\left(\tau \right)R(\tau )\frac{\nabla G_{\xi }\left(\tau \right)}{G_{\xi }\left(\tau \right)}\\ & =\sum_{\tau }G_{\xi }\left(\tau \right)R(\tau )\nabla log(G_{\xi }\left(\tau \right))\\ & \approx \frac{1}{N}\sum_{n=1}^{N}R(\tau_{n})\nabla log(G_{\xi }\left(\tau_{n}\right)) \end{array}$$ (7)

In practice, using the above method will result in a higher variance [64]. To solve this problem, PPO is a more robust algorithm, and the objective function is as follows:

$$\begin{array}{c} J_{PPO}\left(\xi \right)=E\left[min\left(\frac{G_{\xi }\left(\tau \right)}{G_{\hat{\xi }}\left(\tau \right)}R\left(\tau \right),clip\left(\frac{G_{\xi }\left(\tau \right)}{G_{\hat{\xi }}\left(\tau \right)},1-\epsilon ,1+\epsilon \right)R\left(\tau \right)\right)\right] \end{array}$$ (8)

where the $G_{\hat{\xi }}$ is the old policy generator parameterized by $\hat{\xi }$, *R* is the reward of an entire sequence and the *clip* function is defined by *clip*_*ϵ*_(*x*) = min{max{1 − *ϵ*, *x*}, 1 + *ϵ*}. PPO algorithm will constrain the updating range when training the generator, the specialized clipping in the objective function to remove incentives for the new policy to get far from the old policy.

#### Training process

To expedite the generator’s learning of the expert sample distribution during training, pre-training on ChEMBL is imperative. Specifically, the generator is trained on a collection of sequences that encompass standard molecular samples, employing Maximum Likelihood Estimation (MLE) as the loss function. This approach enables the generator to acquire the grammatical attributes of SMILES molecular sequences, thereby ensuring the validity of the generated molecules.

**Algorithm 1** Algorithm of MTMol-GPT.

**Require**: The pre-trained generator *G*_*ξ*_;

**Ensure**: The final trained generator *G*_*ξ*_;

1: Initialize the discriminator *D*_*ϕ*_ and the replay Buffer *B*;

2: **for** *i* = 1 : *maxIter* **do**

3:  Generate sequences *X*_*G*_ by the generator;

4:  Sample sequences $X_{d_{1}}$ and $X_{d_{2}}$ from different demonstration molecules;

5:  Put sequences *X*_*G*_, $X_{d_{1}}$ and $X_{d_{2}}$ into replay Buffer *B*;

6:  Compute the reward of sequences in Buffer *B*;

7:  Update the discriminator *D*_*ϕ*_ with Eq 2c

8:  Update the generator *G*_*ξ*_ using the PPO with Eq 8

9:  Clear Buffer B

10: **end for**

After the pre-training process, we alternately train the discriminator *D*_*ϕ*_ and the generator *G*_*ξ*_ using different demonstration data types. As shown in Fig 7B, we combine the SMILES sequences *X*_*G*_ generated by the pre-trained generator and the sequences $X_{d_{1}},X_{d_{2}}$ sampled from different demonstration molecules. Then, we put the sequences to the replay buffer *B* as the training samples. Next, the same number of sequences generated by the generator and the target molecular sequence is extracted from the replay buffer as the input of the discriminator to predict the generated sequence reward. After that, these predicted rewards are utilized by PPO algorithms to update the generator *G*_*ξ*_, and finally, the cross-entropy loss is used to update the discriminator. Repeat the above steps until the sequences generated by the generator meet the required properties. The process of MTMol-GPT is illustrated in Algorithm 1.

### Implementation details

In our experiments, the generated SMILES sequences were limited to 140 lengths. The dimension of the embedding layer of the generator was set to 128, the dimension of the feed-forward network model was 512, and applied 12-layer decoder with 8 heads. For the discriminator, the embedding size was 128, the feed-forward layer dimension was 256, and a 2-layer bidirectional Gated Recurrent Unit (GRU) network was adopted. Both the generator and discriminator use the Adam [65] to optimize the parameters, and the learning rates were 3 × 10^−5^ and 10^−5^, respectively.

During the process of generator pre-training, the batch size was set to 32, and each epoch required training of all ChEMBL molecules for a total of 500 epochs until the loss was no longer reduced.

Before the GAIL training, it is necessary to perform a warm-up operation on the discriminator to improve the classification accuracy. In each epoch of the training process, the replay buffer collected 512 molecules, of which the real molecules accounted for 0.4 (two target molecules each accounted for 0.2), and the generator and discriminator cyclically extracted 32 molecules from 512 molecules for training. In addition, each time the training generator, PPO algorithm loop once, *ϵ* is set to 0.2. The maximum number of training epoch was set to 1000, and it stopped early during the training process until the loss did not decrease.

## Supporting information

S1 Text Supplementary information file, including supplementary figures A-K and supplementary tables A-D. (PDF)

S1 Data Supplementary information data file. All datasets for each figure and table are structured in the supporting_data.zip file.
(ZIP)
