# Supplementary material for: MTMol-GPT: De novo multi-target molecular generation with transformer-based generative adversarial imitation learning
Source: PLoS Comput Biol. 2024 Jun 26;20(6):e1012229. doi: 10.1371/journal.pcbi.1012229 (PMC11233020; doi:10.1371/journal.pcbi.1012229)
Supplement: S1 Text — (PDF) [file pcbi.1012229.s001.pdf]

# MTMol-GPT: De novo multi-target molecular generation with transformer-based generative adversarial imitation learning

Chengwei Ai[1], Hongpeng Yang[2], Xiaoyi Liu[2], Ruihan Dong[3], Yijie Ding[4*], Fei Guo[1*]

**1** School of computer science and engineering, Central South University, Changsha, China
**2** Department of computer science and engineering, University of South Carolina, Columbia, USA
**3** Academy for Advanced Interdisciplinary Studies, Chinese Academy of Sciences, Peking University, Beijing, China
**4** Yangtze Delta Region Institute (Quzhou), University of Electronic Science and Technology of China, Quzhou, China.

## Supporting information

### Comparison of our method with sota methods in single target task.

In order to demonstrate that the model is effective not only on dual-target molecules but also on single-target molecules. We compared the SOTA models on single target molecule generation, which are LS-MolGen and cMolGPT. LS-MolGen divides the training steps into three steps. The first step is to train a generative network on a large dataset to ensure that the trained generative network has the ability to generate valid molecules. The second step adopts transfer learning to train on the desired target dataset, allowing the model to generate the potential of current target molecules; The third step of the paper adopts reinforcement learning methods, using Docking score as a reward to optimize the generator and generate molecules with high target affinity. cMolGPT utilizes GPT models to achieve molecule generation on specific targets, which implement different keys and values for the multi-head attention conditional on a specified target.

From the Tables A and B results above, it can be seen that compared to the state-of-the-art methods, our SMILES based method has also achieved the lowest of FCD value. In addition, our SELFIES based method is superior to the state-of-the-art method in terms of valid and internal diversity (IntDiv). In summary, our model has advantages in generating single targets drug like molecules in addition to dual targets.

### Evaluating the pre-trained model

The pre-training process plays a crucial role in our model. In the initial experiment, the pre-trained model was utilized without fine-tuning to generate a dataset comprising $10,000$ valid molecules. The properties (QED, LogP, and SA score) of these molecules were evaluated using the ChEMBL dataset. A comparative analysis of the property distributions between the generated molecules and the ChEMBL molecules highlights the significance of pre-training in comprehensively understanding the property distributions of well-established molecules.

| Metrics | cMolGPT | LS-MolGen | MTMol-GPT | SF-MTMol-GPT |
|---|---|---|---|---|
| valid | 0.8891 | 0.9453 | 0.8700 | **1.0000** |
| unique@1000 | 0.9910 | **1.0000** | 0.9890 | 0.9380 |
| unique@10000 | 0.9593 | **0.9997** | 0.9345 | 0.7970 |
| Novelty | **0.9932** | 0.9864 | 0.9870 | 0.9668 |
| IntDiv | 0.8366 | 0.8519 | 0.8405 | **0.8557** |
| *FCD/(DRD2) | 5.6133 | 6.5425 | **4.8212** | 5.5787 |
| SNN/(DRD2) | 0.4247 | 0.3890 | 0.4346 | **0.4385** |
| Frag/(DRD2) | 0.9726 | 0.9749 | **0.9818** | 0.9547 |
| Scaf/(DRD2) | 0.5634 | 0.5619 | 0.6553 | **0.6827** |
| *FCD/(HTR1A) | 8.813 | 8.6870 | **8.5154** | 10.2562 |
| SNN/(HTR1A) | **0.3744** | 0.3467 | 0.3711 | 0.3386 |
| Frag/(HTR1A) | **0.8906** | 0.8656 | 0.8830 | 0.8139 |
| Scaf/(HTR1A) | 0.1877 | 0.2032 | **0.2141** | 0.1345 |

* denotes that the lower score in this metric is better. Bolded values denote the best performance for each metric.

**Table A.** Comparison of Our method with cMolGPT and LS-MolGen in single target task (DRD2) by using MOSES metrics.

| Metrics | cMolGPT | LS-MolGen | MTMol-GPT | SF-MTMol-GPT |
|---|---|---|---|---|
| valid | 0.8695 | 0.9198 | 0.8089 | **1.0000** |
| unique@1000 | 0.9940 | **1.0000** | 0.9900 | 0.9620 |
| unique@10000 | 0.9537 | **0.9998** | 0.9462 | 0.8328 |
| Novelty | **0.9876** | 0.9839 | **0.9887** | 0.9683 |
| IntDiv | 0.8233 | 0.8448 | 0.8390 | **0.8550** |
| FCD/(DRD2) | 8.7298 | 8.9651 | **8.5087** | 10.3727 |
| SNN/(DRD2) | **0.3807** | 0.3510 | 0.3574 | **0.3251** |
| Frag/(DRD2) | 0.9305 | **0.9508** | **0.9443** | 0.9175 |
| Scaf/(DRD2) | 0.1217 | 0.1579 | 0.1502 | **0.1782** |
| FCD/(HTR1A) | 4.9061 | 5.3286 | **4.7907** | 5.8430 |
| SNN/(HTR1A) | **0.4454** | 0.4049 | 0.4192 | 0.4259 |
| Frag/(HTR1A) | **0.9782** | 0.9721 | 0.9654 | 0.9061 |
| Scaf/(HTR1A) | 0.3364 | 0.4175 | **0.4100** | **0.5660** |

* denotes that the lower score in this metric is better. Bolded values denote the best performance for each metric.

**Table B.** Comparison of Our method with cMolGPT and LS-MolGen in single target task (HTR1A) by using MOSES metrics.

The visual representation in Fig A provides a clear depiction of the distributions of the generated molecules. The close alignment between these distributions and the ChEMBL dataset underscores the exceptional ability of the pre-trained MTMol-GPT model to capture the inherent properties of ChEMBL molecules. Moreover, the convergence observed in the QED and LogP distributions with SF-MTMol-GPT further emphasizes its efficacy. However, it should be noted that the distribution of the SA score deviates discernibly from the distributions observed in the ChEMBL dataset.

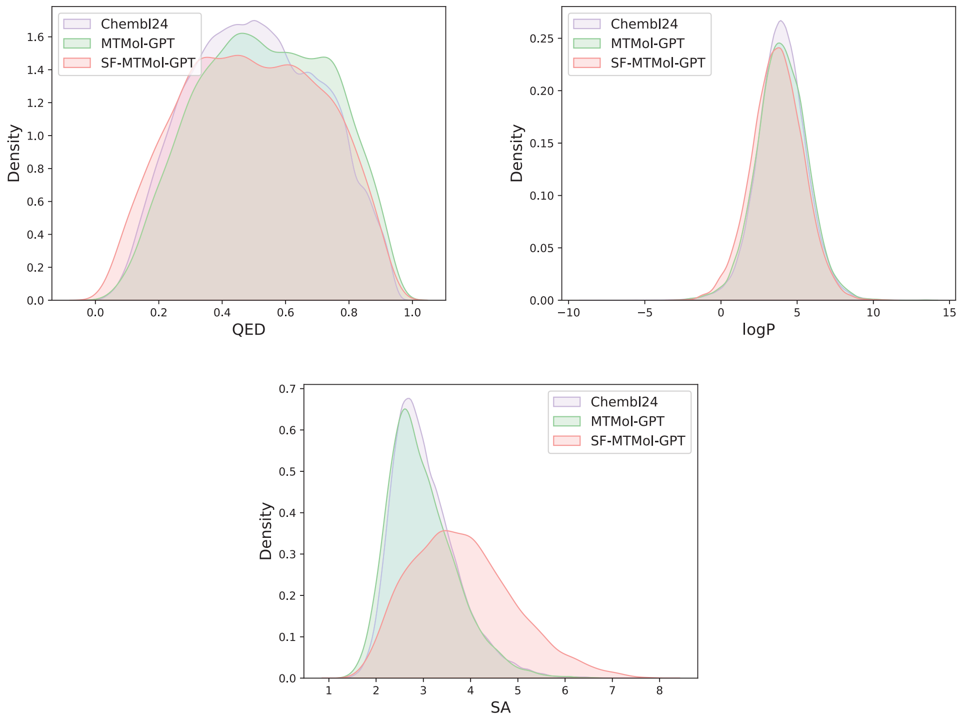


**Fig A. Distributions of molecular properties with pre-trained model and ChEMBL molecules.** QED, LogP, and SA score distributions for ChEMBL molecules, and molecules generated by the pre-treained MTMol-GPT and SF-MTMol-GPT.

## Visualization of generated molecules

In this section, we visualize the molecules generated from the pre-trained and fine-tuned models, as exhibited in Figs B-E. This illustrative representation underscores that the produced molecules possess the requisite structural integrity alongside a commendable QED score, indicative of their high potential for practical application.

To present an unequivocal and exhaustive interpretation of our study, we have constructed sampled molecules emanating from pre-trained and fine-tuned models. These visual narratives yield invaluable perspectives on the molecular architecture birthed under diverse modeling conditions, underscoring the adaptability and efficacy of our methodologies. Moreover, we have curated a captivating compilation of molecules generated through our models, originating from both SMILES and SELFIES depictions. These graphic renditions not only confirm the robustness inherent to our model but also accentuate the potential relevance of these molecules in

practical applications.

The combination of our findings presents a compelling narrative, highlighting the model's exceptional ability to generate diverse and valid molecular structures. The visual representation, coupled with detailed analysis, provides a comprehensive overview, paving the way for future exploration in the exciting field of cheminformatics and de novo drug design.

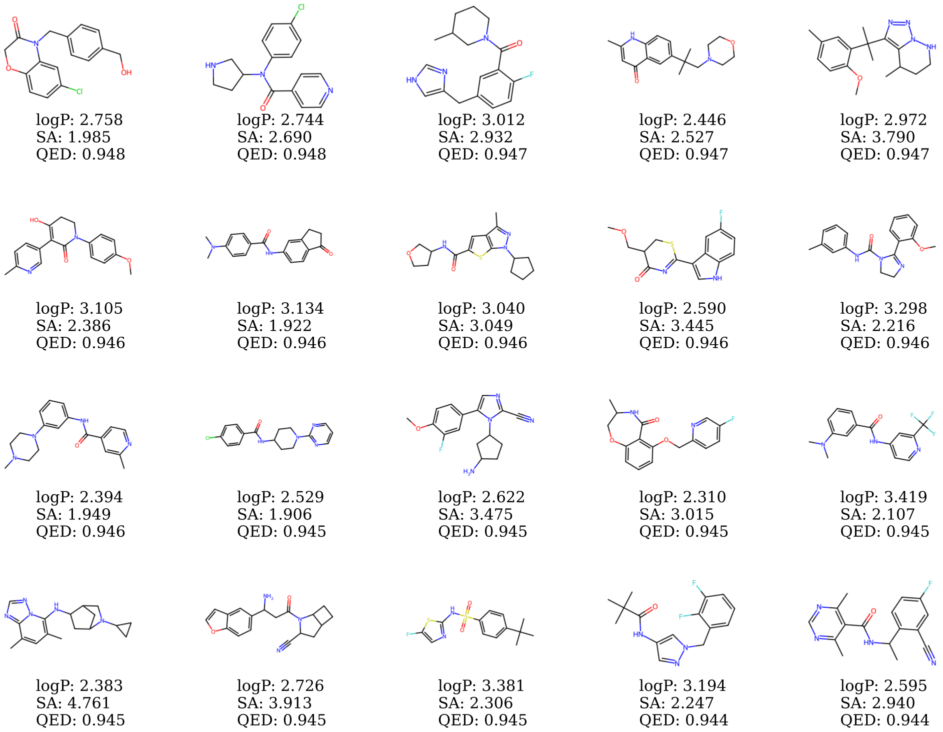


**Fig B.** Molecules generated from the pre-trained model based on SMILES.

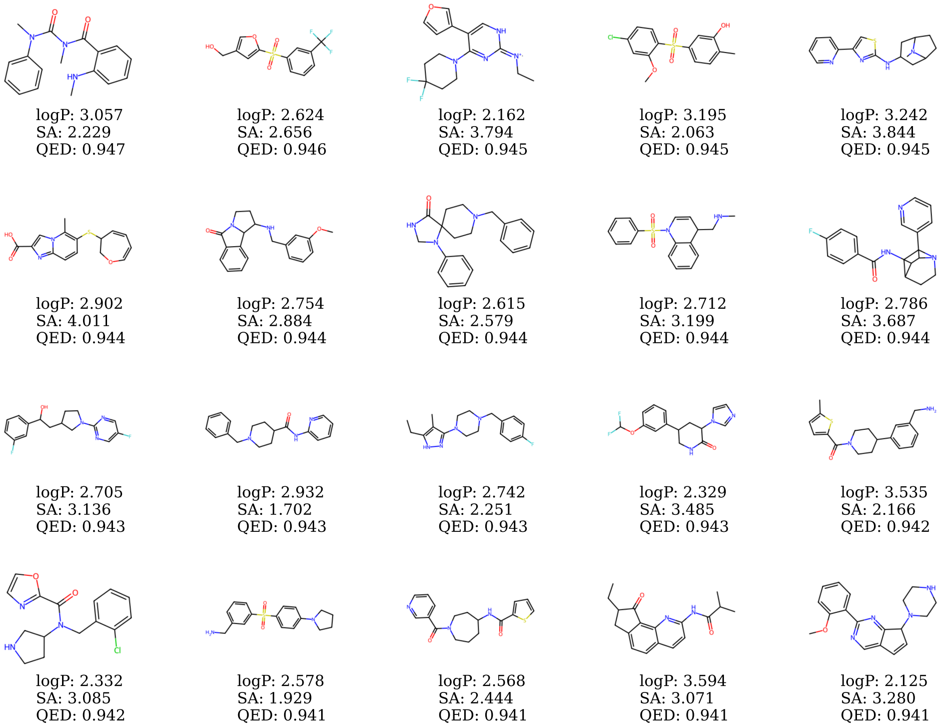


**Fig C.** Molecules generated from the pre-trained model based on SELFIES.

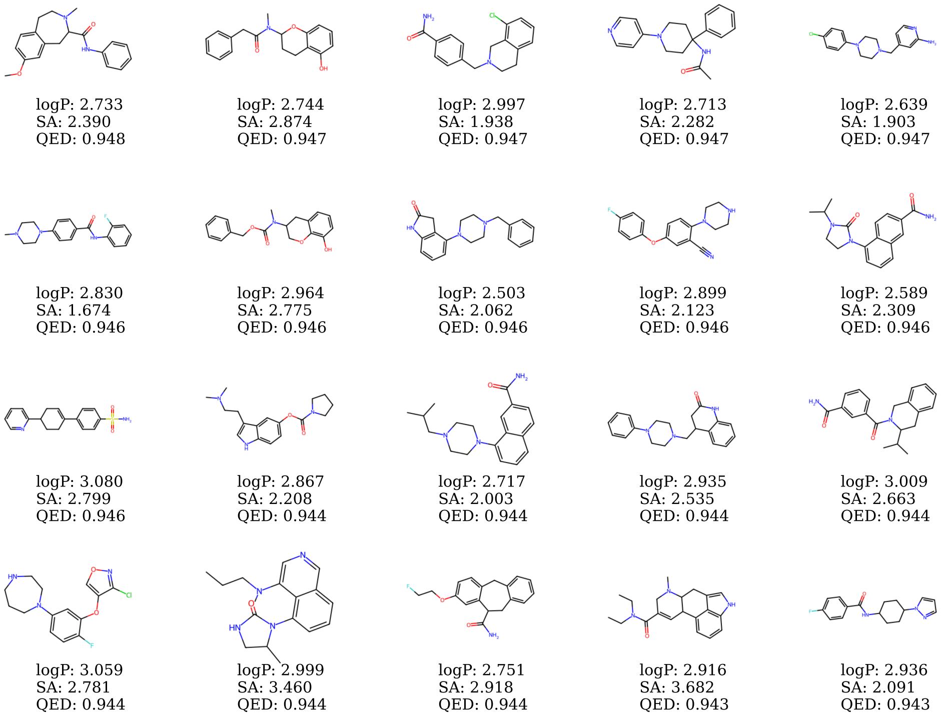


**Fig D.** Molecules generated from the fine-tuned model based on SMILES.

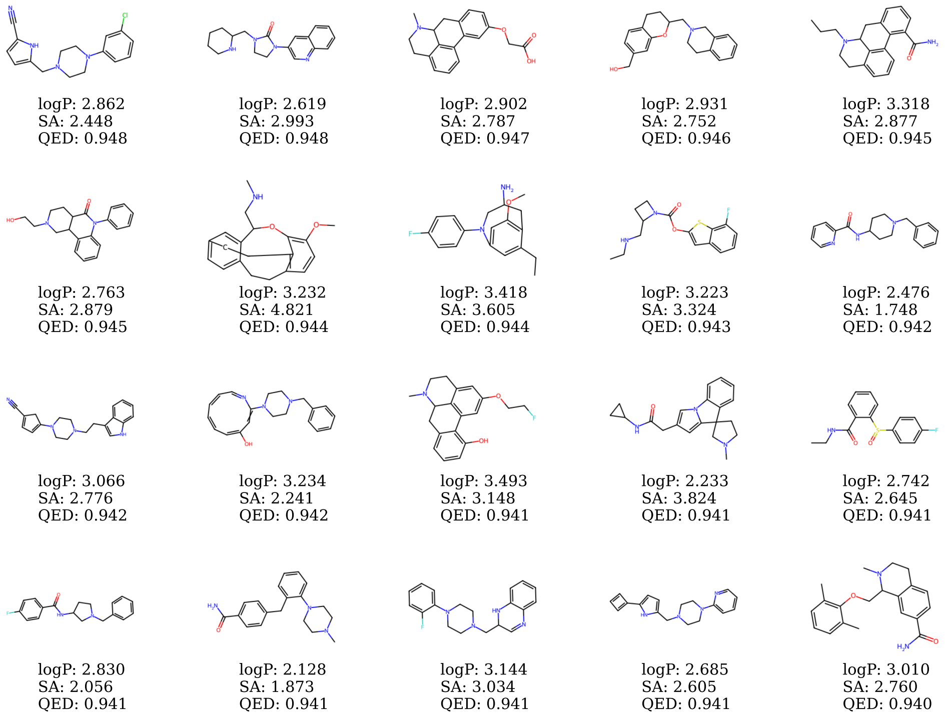


**Fig E.** Molecules generated from the fine-tuned model based on SELFIES.

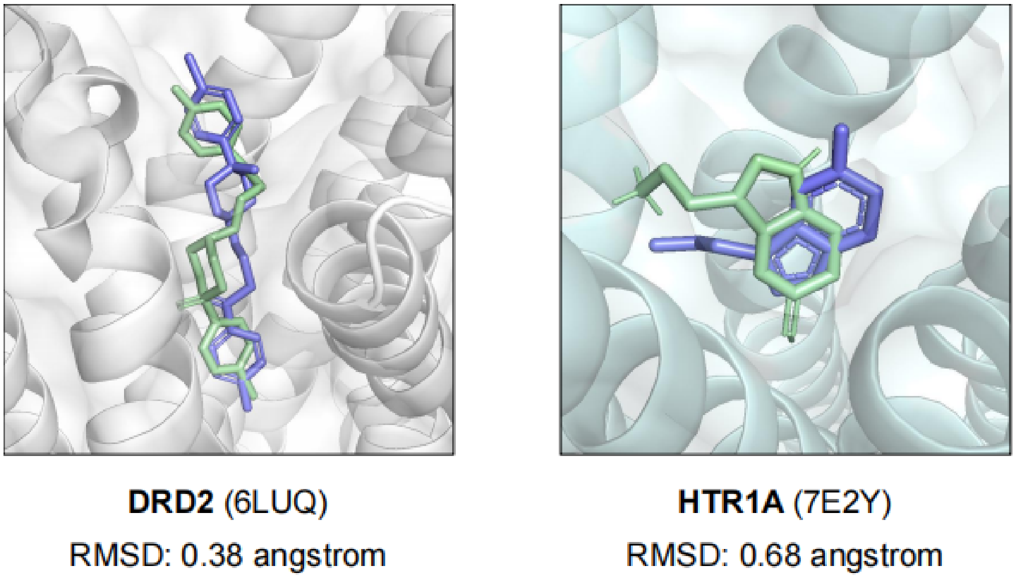


**Fig F. Redocking validation of DRD2 and HTR1A targets.** Ligands in native structure are shown in purple, and redocked conformations are in palegreen.

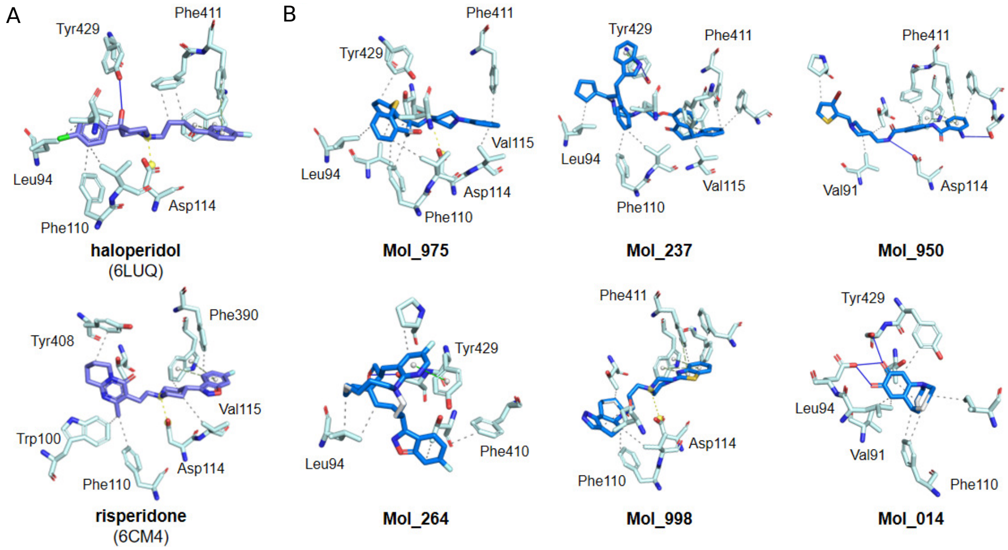


**Fig G. Protein-ligand binding interactions of ligands with DRD2.** (a) crystal complexes (PDB: 6LUQ, 6CM4). (b) MTMol-GPT generated representative molecules docked with DRD2.

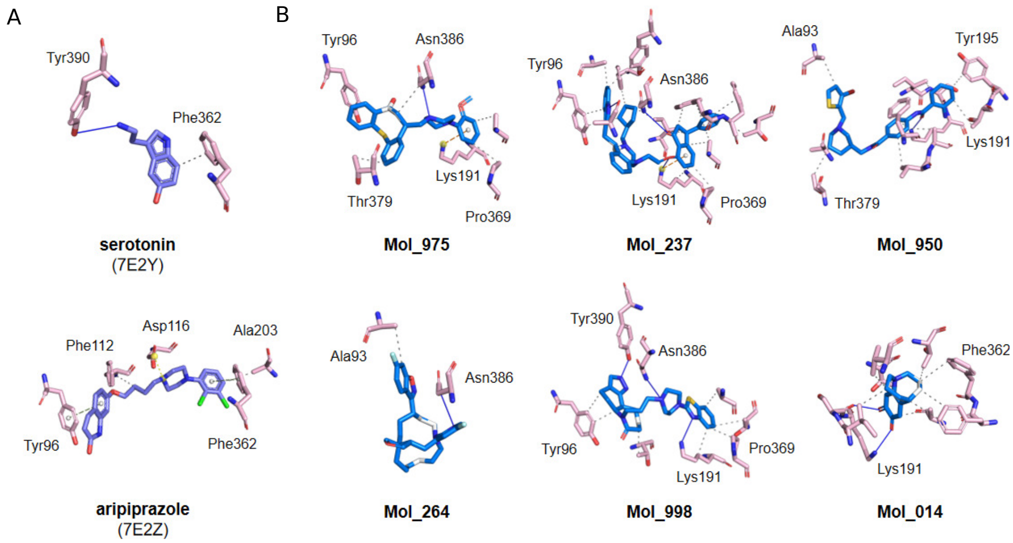


**Fig H. Protein-ligand binding interactions of ligands with HTR1A.** (a) crystal complexes (PDB: 7E2Y, 7E2Z). (b) MTMol-GPT generated representative molecules docked with HTR1A.

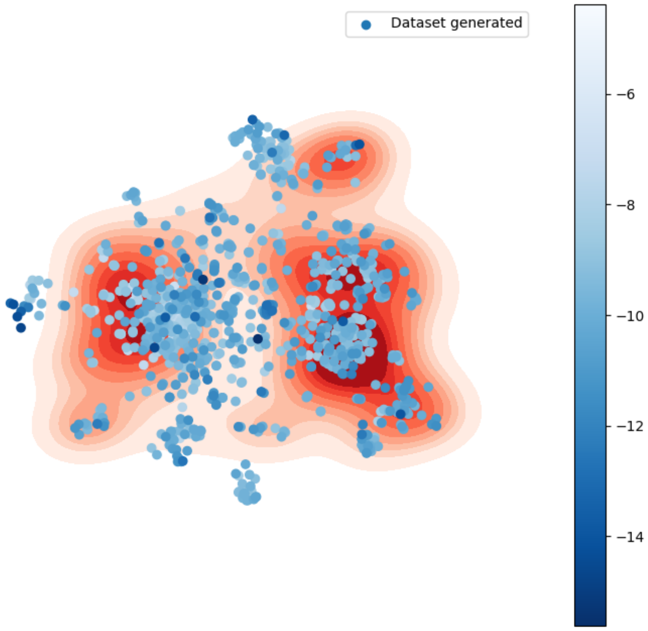


**Fig I.** The t-SNE visualization of generated and EGFR test set molecules fingerprints.

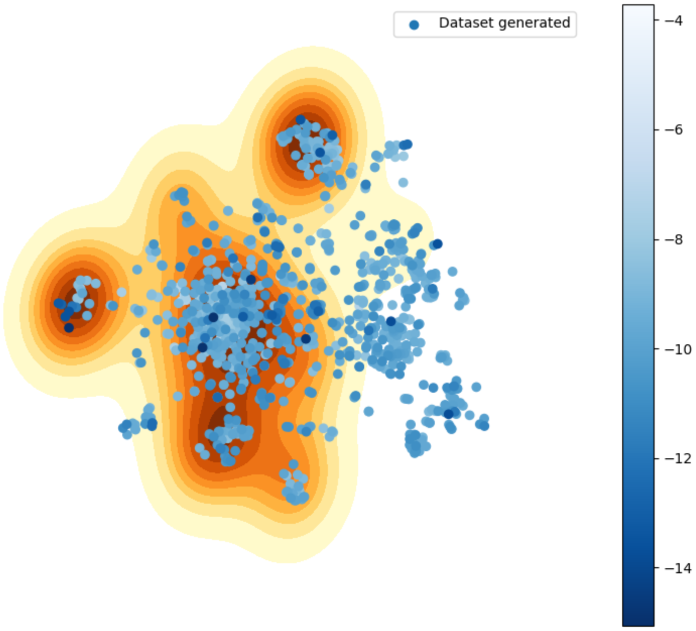


**Fig J.** The t-SNE Visualization of Generateda and SRC test set Molecules Fingerprints.

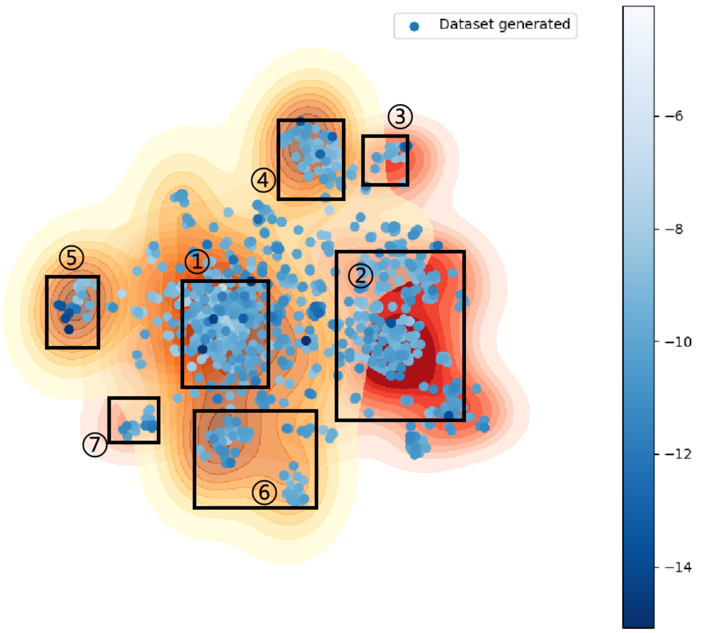


**Fig K.** The t-SNE Visualization of Generated, EGFR test set and SRC test set Molecules Fingerprints.

| Region | Index | SMILES | DRD2_Ds | HTR1A_Ds | logp | QED | SA |
|---|---|---|---|---|---|---|---|
| 1 | 975 | COc1ccccc1N1CCN(CC2CC(=O)c3ccccc3Sc3ccccc32)CC1 | -15.180 | -12.920 | 5.3386 | 0.5425 | 2.8417 |
| 2 | 237 | O=C1CC(c2ccccc2)c2cccc(OCCN3CCN(CC4CCCC4)CC(Cc4c[nH] c5ccccc45)c4ccccc43)c21 | -13.770 | -14.750 | 8.6037 | 0.1787 | 3.6983 |
| 3 | 950 | Nc1ccccc1C(=O)Nc1ccc(C(=O)NCC2CCCN(Cc3sccc3Br)C2)cc1 | -10.420 | -9.952 | 4.9871 | 0.3783 | 2.7650 |
| 4 | 264 | Fc1cnc2c(c1)CC1COCC13CCCCCCN2CCC(c1noc2cc(F)ccc12)CC3 | -14.950 | -14.900 | 6.4145 | 0.4017 | 5.2919 |
| 5 | 998 | O=C1CCCC2c3n[nH]cc3CCC2N1CCCN1CCN(c2nc3ccccc3s2)CC1 | -14.030 | -13.510 | 3.6426 | 0.6244 | 3.6652 |
| 6 | 14 | Oc1cc2c(cc1O)N1CCCC2CCC1 | -15.500 | -15.810 | 2.5754 | 0.6590 | 2.9147 |

**Table C.** Molecular information about DRD2 and HTR1A target in different regions (Ds: docking score).

| Region | Index | SMILES | EGFR_Ds | SRC_Ds | logp | QED | SA |
|---|---|---|---|---|---|---|---|
| 1 | 914 | CC(=O)Oc1cccc2c1C(C(=O)Nc1ccccc1)=C1SCC2NCCOCCN1CCc1ccccc1 | -13.740 | -13.1600 | 0.3392 | 4.8716 | 4.4873 |
| 2 | 6 | CN(C)CCN(C)Cc1c[nH]c2cc3ncnc(Nc4cccc(Br)c4)c3cc12 | -8.694 | -9.143 | 0.4281 | 4.6106 | 2.5770 |
| 3 | 815 | CCOc1cc2ncc(C#N)c(Nc3ccc(O[C@H](C)c4ccccc4)c(Cl)c3)c2cc1NC(=O)/C=C/CN1CCCOCC1 | -12.730 | -11.280 | 0.1677 | 7.2592 | 3.4190 |
| 4 | 306 | COc1cc(Nc2c(C#N)cnc3cc(-c4cccc(CN5CCNS(=O)CC5)n4)ccc23)c(Cl)cc1Cl | -12.160 | -12.170 | 0.3262 | 5.2964 | 3.6214 |
| 5 | 389 | O=C(N[C@H]1CCCCN(Cc2ccc(O[PH](=O)(=O)c3ccccc3)cc2)C1=O)c1ccc(-c2ccccc2)cc1 | -13.310 | -13.690 | 0.2812 | 5.7249 | 3.3980 |
| 6 | 319 | COc1cc(-c2nn(C3CCC(N4CCN(C)CC4)CC3)c3ncnc(N)c23)ccc1NC(=O)c1cc2ccccc2[nH]1 | -11.500 | -9.932 | 0.2672 | 4.5488 | 2.9032 |
| 7 | 361 | Cc1ccc(C2=NN(C3=NC(c4ccccc4)CS3)C(c3cccc4ccccc34)C2)[nH]1 | -10.4200 | -11.2200 | 0.3998 | 6.4715 | 3.6489 |

* Region 1 is the overlapping region, regions 2 and 7 are the concentrated regions of EGFR test set molecules, and regions 3, 4, 5, and 6 are the concentrated regions of SRC target test set molecules.

**Table D.** Molecular information about EGFR and SRC target in different regions, Docking score (Ds).
