# Supplementary material for: MTMol-GPT: De novo multi-target molecular generation with transformer-based generative adversarial imitation learning
Source: PLoS Comput Biol. 2024 Jun 26;20(6):e1012229. doi: 10.1371/journal.pcbi.1012229 (PMC11233020; doi:10.1371/journal.pcbi.1012229)
Supplement: S1 Data — All datasets for each figure and table are structured in the supporting_data.zip file. (ZIP) [file pcbi.1012229.s002.zip › data/figS1-S11/S_figureS2-S5/results/sf_results/selfies_e0_supp.pdf]

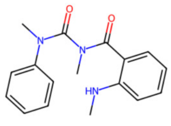

logP: 3.057
SA: 2.229
QED: 0.947

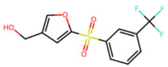

logP: 2.624
SA: 2.656
QED: 0.946

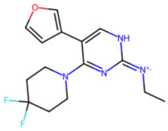

logP: 2.162
SA: 3.794
QED: 0.945

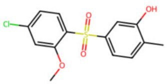

logP: 3.195
SA: 2.063
QED: 0.945

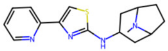

logP: 3.242
SA: 3.844
QED: 0.945

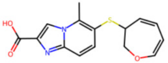

logP: 2.902
SA: 4.011
QED: 0.944

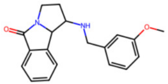

logP: 2.754
SA: 2.884
QED: 0.944

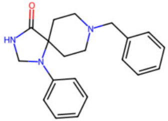

logP: 2.615
SA: 2.579
QED: 0.944

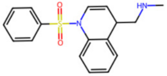

logP: 2.712
SA: 3.199
QED: 0.944

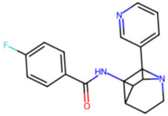

logP: 2.786
SA: 3.687
QED: 0.944

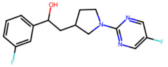

logP: 2.705
SA: 3.136
QED: 0.943

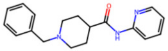

logP: 2.932
SA: 1.702
QED: 0.943

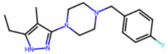

logP: 2.742
SA: 2.251
QED: 0.943

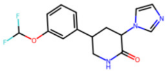

logP: 2.329
SA: 3.485
QED: 0.943

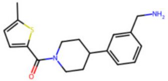

logP: 3.535
SA: 2.166
QED: 0.942

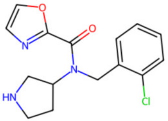

logP: 2.332
SA: 3.085
QED: 0.942

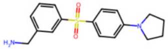

logP: 2.578
SA: 1.929
QED: 0.941

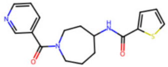

logP: 2.568
SA: 2.444
QED: 0.941

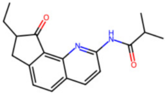

logP: 3.594
SA: 3.071
QED: 0.941

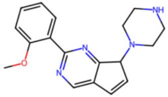

logP: 2.125
SA: 3.280
QED: 0.941
