# Supplementary material for: MTMol-GPT: De novo multi-target molecular generation with transformer-based generative adversarial imitation learning
Source: PLoS Comput Biol. 2024 Jun 26;20(6):e1012229. doi: 10.1371/journal.pcbi.1012229 (PMC11233020; doi:10.1371/journal.pcbi.1012229)
Supplement: S1 Data — All datasets for each figure and table are structured in the supporting_data.zip file. (ZIP) [file pcbi.1012229.s002.zip › data/figS1-S11/S_figureS2-S5/results/sf_results/selfies_e190_supp.pdf]

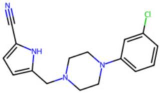

logP: 2.862
SA: 2.448
QED: 0.948

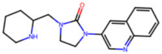

logP: 2.619
SA: 2.993
QED: 0.948

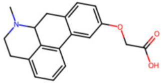

logP: 2.902
SA: 2.787
QED: 0.947

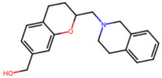

logP: 2.931
SA: 2.752
QED: 0.946

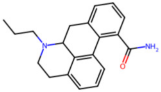

logP: 3.318
SA: 2.877
QED: 0.945

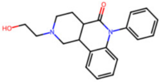

logP: 2.763
SA: 2.879
QED: 0.945

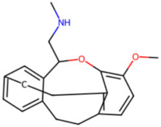

logP: 3.232
SA: 4.821
QED: 0.944

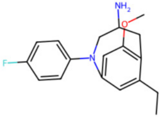

logP: 3.418
SA: 3.605
QED: 0.944

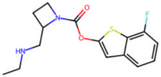

logP: 3.223
SA: 3.324
QED: 0.943

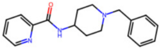

logP: 2.476
SA: 1.748
QED: 0.942

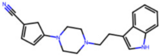

logP: 3.066
SA: 2.776
QED: 0.942

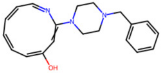

logP: 3.234
SA: 2.241
QED: 0.942

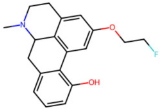

logP: 3.493
SA: 3.148
QED: 0.941

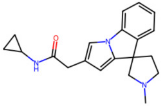

logP: 2.233
SA: 3.824
QED: 0.941

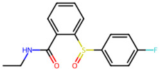

logP: 2.742
SA: 2.645
QED: 0.941

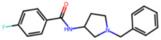

logP: 2.830
SA: 2.056
QED: 0.941

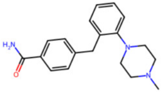

logP: 2.128
SA: 1.873
QED: 0.941

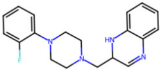

logP: 3.144
SA: 3.034
QED: 0.941

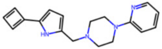

logP: 2.685
SA: 2.605
QED: 0.941

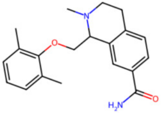

logP: 3.010
SA: 2.760
QED: 0.940
