# Supplementary material for: MTMol-GPT: De novo multi-target molecular generation with transformer-based generative adversarial imitation learning
Source: PLoS Comput Biol. 2024 Jun 26;20(6):e1012229. doi: 10.1371/journal.pcbi.1012229 (PMC11233020; doi:10.1371/journal.pcbi.1012229)
Supplement: S1 Data — All datasets for each figure and table are structured in the supporting_data.zip file. (ZIP) [file pcbi.1012229.s002.zip › data/figS1-S11/S_figureS2-S5/results/sm_results/smiles_e0_supp.pdf]

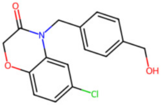

logP: 2.758
SA: 1.985
QED: 0.948

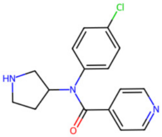

logP: 2.744
SA: 2.690
QED: 0.948

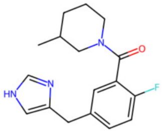

logP: 3.012
SA: 2.932
QED: 0.947

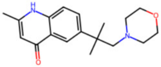

logP: 2.446
SA: 2.527
QED: 0.947

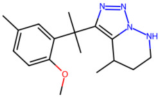

logP: 2.972
SA: 3.790
QED: 0.947

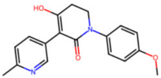

logP: 3.105
SA: 2.386
QED: 0.946

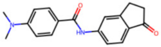

logP: 3.134
SA: 1.922
QED: 0.946

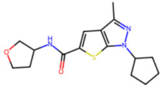

logP: 3.040
SA: 3.049
QED: 0.946

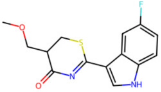

logP: 2.590
SA: 3.445
QED: 0.946

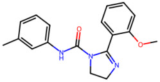

logP: 3.298
SA: 2.216
QED: 0.946

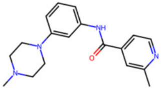

logP: 2.394
SA: 1.949
QED: 0.946

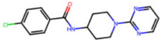

logP: 2.529
SA: 1.906
QED: 0.945

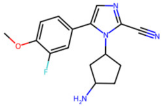

logP: 2.622
SA: 3.475
QED: 0.945

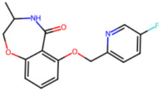

logP: 2.310
SA: 3.015
QED: 0.945

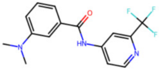

logP: 3.419
SA: 2.107
QED: 0.945

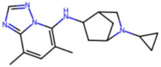

logP: 2.383
SA: 4.761
QED: 0.945

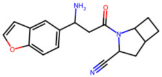

logP: 2.726
SA: 3.913
QED: 0.945

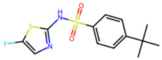

logP: 3.381
SA: 2.306
QED: 0.945

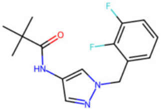

logP: 3.194
SA: 2.247
QED: 0.944

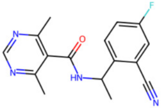

logP: 2.595
SA: 2.940
QED: 0.944
