# Supplementary material for: MTMol-GPT: De novo multi-target molecular generation with transformer-based generative adversarial imitation learning
Source: PLoS Comput Biol. 2024 Jun 26;20(6):e1012229. doi: 10.1371/journal.pcbi.1012229 (PMC11233020; doi:10.1371/journal.pcbi.1012229)
Supplement: S1 Data — All datasets for each figure and table are structured in the supporting_data.zip file. (ZIP) [file pcbi.1012229.s002.zip › data/figS1-S11/S_figureS2-S5/results/sm_results/smiles_e30_supp.pdf]

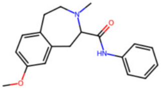

logP: 2.733
SA: 2.390
QED: 0.948

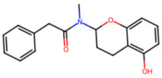

logP: 2.744
SA: 2.874
QED: 0.947

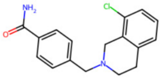

logP: 2.997
SA: 1.938
QED: 0.947

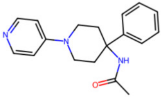

logP: 2.713
SA: 2.282
QED: 0.947

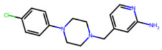

logP: 2.639
SA: 1.903
QED: 0.947

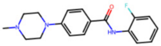

logP: 2.830
SA: 1.674
QED: 0.946

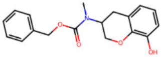

logP: 2.964
SA: 2.775
QED: 0.946

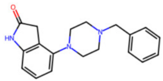

logP: 2.503
SA: 2.062
QED: 0.946

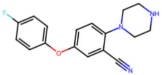

logP: 2.899
SA: 2.123
QED: 0.946

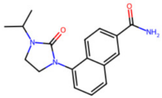

logP: 2.589
SA: 2.309
QED: 0.946

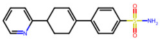

logP: 3.080
SA: 2.799
QED: 0.946

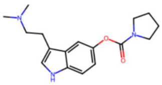

logP: 2.867
SA: 2.208
QED: 0.944

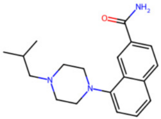

logP: 2.717
SA: 2.003
QED: 0.944

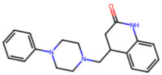

logP: 2.935
SA: 2.535
QED: 0.944

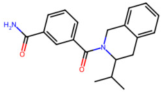

logP: 3.009
SA: 2.663
QED: 0.944

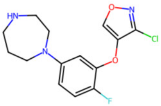

logP: 3.059
SA: 2.781
QED: 0.944

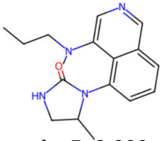

logP: 2.999
SA: 3.460
QED: 0.944

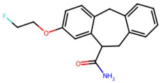

logP: 2.751
SA: 2.918
QED: 0.944

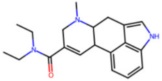

logP: 2.916
SA: 3.682
QED: 0.943

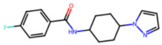

logP: 2.936
SA: 2.091
QED: 0.943
